# From Morphology to Multi-Omics: A New Age of Fusarium Research

**DOI:** 10.3390/pathogens14080762

**Published:** 2025-08-01

**Authors:** Collins Bugingo, Alessandro Infantino, Paul Okello, Oscar Perez-Hernandez, Kristina Petrović, Andéole Niyongabo Turatsinze, Swarnalatha Moparthi

**Affiliations:** 1 Crop and Soil Science Department, Oregon State University, Corvallis, OR 97331, USA; 2 School of Integrative Plant Science, Horticulture Department, Cornell Agritech, Cornell University, Geneva, NY 14456, USA; 3 Council for Agricultural Research and Economics, Research Centre for Plant Protection and Certification of Rome, Via C.G. Bertero 22, 00156 Rome, Italy; alessandro.infantino@crea.gov.it; 4 School of Agricultural Sciences, Northwest Missouri State University, Maryville, MO 64468, USA; pokello@nwmissouri.edu (P.O.); ohernandez@nwmissouri.edu (O.P.-H.); 5 Maize Research Institute “Zemun Polje”, Slobodana Bajića 1, 11000 Belgrade, Serbia; kristina.petrovic@mrizp.rs; 6 State Key Laboratory of Ecological Safety and Sustainable Development in Arid Lands, Northwest Institute of Eco-Environment and Resources, Chinese Academy of Sciences, Lanzhou 730000, China; andeoleniyo2021@gmail.com; 7 Department of Entomology and Plant Pathology, North Carolina State University, Raleigh, NC 27695-7613, USA; smopart@ncsu.edu

**Keywords:** *Fusarium*, species complex, host specificity, mycotoxins, functional genomics, phylogenomics

## Abstract

The *Fusarium* genus includes some of the most economically and ecologically impactful fungal pathogens affecting global agriculture and human health. Over the past 15 years, rapid advances in molecular biology, genomics, and diagnostic technologies have reshaped our understanding of *Fusarium* taxonomy, host–pathogen dynamics, mycotoxin biosynthesis, and disease management. This review synthesizes key developments in these areas, focusing on agriculturally important *Fusarium* species complexes such as the *Fusarium oxysporum* species complex (FOSC), *Fusarium graminearum* species complex (FGSC), and a discussion on emerging lineages such as *Neocosmospora*. We explore recent shifts in species delimitation, functional genomics, and the molecular architecture of pathogenicity. In addition, we examine the global burden of *Fusarium*-induced mycotoxins by examining their prevalence in three of the world’s most widely consumed staple crops: maize, wheat, and rice. Last, we also evaluate contemporary management strategies, including molecular diagnostics, host resistance, and integrated disease control, positioning this review as a roadmap for future research and practical solutions in Fusarium-related disease and mycotoxin management. By weaving together morphological insights and cutting-edge multi-omics tools, this review captures the transition into a new era of Fusarium research where integrated, high-resolution approaches are transforming diagnosis, classification, and management.

## 1. *Fusarium*: A Genus of Global Agricultural and Phytopathological Significance

*Fusarium* is a cosmopolitan genus of *Ascomycota* (class *Sordariomycetes*, order *Hypocreales*, family *Nectriaceae*), characterized by high species diversity and considerable economic impact across global agriculture [1] comprising over 400 phylogenetically distinct species, many of which form species complexes with overlapping ecological and pathogenic roles [2]. These species are organized into at least 22 species complexes, with additional monotypic lineages identified through molecular phylogenetic analyses [3]. This genus comprises saprophytic, endophytic, and pathogenic species responsible for a range of plant diseases, including vascular wilts, root rots, and head blights [4]. *Fusarium* spp. infects a broad range of hosts, including cereals, legumes, and horticultural crops, and are frequently implicated in postharvest decay [5,6,7,8]. *Fusarium graminearum* and *F. oxysporum* are consistently ranked among the top ten fungal pathogens of plants globally due to their host range, virulence, and economic impact [9]. Many *Fusarium* species are also known to produce fusariotoxins, which include secondary metabolites such as trichothecenes, fumonisins, and zearalenone. These compounds can cause toxic effects in humans and animals upon consumption of contaminated grains [10].

*Fusarium*-induced symptoms are highly variable and often non-specific, limiting the reliability of visual diagnosis. Symptomatology includes wilting, vascular discoloration, chlorosis, and rots affecting the root, crown, stem or seed, with variation depending on host and *Fusarium* species [8,11,12,13]. Symptoms overlap with other seed- and soilborne pathogens, or abiotic stressors, further complicates field-level diagnosis. While pathogen isolation and culturing remain foundational, morphological differentiation is hampered by shared features such as colony pigmentation, conidial morphology, and growth rate. Moreover, species within the *F. oxysporum* species complex (FOSC) or the *F. solani* species complex (FSSC) may show minimal morphological differences while displaying significant variation in pathogenicity and host range [4,14]. Consequently, molecular techniques, including species-specific PCR assays, sequencing of conserved genomic regions, and whole-genome comparisons, have become essential tools for accurate identification and classification of *Fusarium* species in diagnostic and research settings [15].

The taxonomy of *Fusarium* remains dynamic, driven by the ongoing discovery of novel taxa and reevaluation of existing lineages through molecular approaches [2]. Recent tools such as FusaHelp have augmented traditional morphological identification by providing web-based platforms for rapid species comparison [16]. Regardless of morphological features, Infantino et al. [16] pointed out that *F. equiseti* and *F. compactum* exhibit very similar morphological characteristics when cultured on artificial media, highlighting that molecular markers are crucial for the accurate identification of these microorganisms. Multilocus phylogenetics, DNA barcoding, and genome-scale approaches have redefined *Fusarium* systematics, enabling higher-resolution species delimitation [17]. As of this writing, the number of phylogenetically supported *Fusarium* species continues to rise, and with it, the complexity of species recognition and nomenclature.

Anthropogenic drivers such as climate change, international trade, and intensive monoculture are expected to exacerbate the spread and impact of *Fusarium* diseases [18]. Given recent advancements in taxonomy, diagnostics, and functional genomics, an updated synthesis is warranted to contextualize emerging patterns and inform research priorities. This review integrates developments across five key domains: (1) taxonomy and phylogenetics, (2) genomic and functional analyses, (3) host–pathogen interactions, (4) mycotoxin biosynthesis and toxicology, and (5) disease management. This review mirrors the trajectory from classical morphological identification to advanced multi-omics strategies, showcasing how genomics, transcriptomics, and functional assays are reshaping our understanding of *Fusarium*’s taxonomy, pathogenicity, and ecology. The goal is to offer an interdisciplinary framework for understanding *Fusarium* biology and guiding future directions in research and management.

## 2. Taxonomy and Phylogenetics of *Fusarium* Species

The classification of Fusarium has undergone a profound transformation [19,20,21,22,23,24], shifting from traditional morphology-based taxonomy to advanced molecular approaches, including multilocus sequencing and genome-wide analyses. This evolution has been instrumental in recognizing the complexity of species groups, such as FOSC, Fusarium fujikuroi species complex (FFSC), FGSC, and Fusarium incarnatum-equiseti species complex (FIESC), which play significant roles in agriculture and plant pathology. The taxonomic debate surrounding Neocosmospora has further reshaped perspectives, raising questions about whether its reclassification reflects a broader genus redefinition. Additionally, newly emerging Fusarium species are gaining attention due to their increasing impact on niche crops in diverse geographic regions. Recent phylogenetic insights drawn from whole-genome datasets continue to refine our understanding of species boundaries, host specificity, and evolutionary trajectories, underscoring the relevance of molecular tools in fungal systematics.

### 2.1. From Morphology to Genomics: Evolution of Fusarium Classification

The taxonomy of *Fusarium* has undergone significant transformation over the past century, transitioning from traditional morphological classification to a multifaceted taxonomic framework incorporating molecular techniques, reflecting the increasing need for accurate pathogen identification in agricultural and ecological settings. Initially, species identification relied heavily on morphological characteristics based on observable phenotypic traits, such as conidial shape, septation, and pigmentation, as detailed in the foundational works of Buxton [19], Snyder & Hansen [20], and Booth [21]. However, these phenotypic traits often exhibited plasticity and convergence, leading to misidentifications and taxonomic ambiguities, particularly in delineating species within this genetically and ecologically diverse genus. *Fusarium* taxonomy has undergone multiple revisions to address these challenges, moving from traditional classification based on morphological traits to DNA sequence-based approaches. Multilocus sequence typing (MLST) using genes such as translation elongation factor 1-alpha (TEF1-α), RNA polymerase II subunits (RPB1 and RPB2), and β-tubulin (TUB2) has enhanced species resolution and facilitated the recognition of cryptic species complexes [22]. Despite these advances, challenges persist due to cryptic species and morphological plasticity, as well as frequent horizontal gene transfer and hybridization events that blur species boundaries [23]. Recent genome-wide phylogenomic approaches have provided deeper insights into the evolutionary relationships within the genus *Fusarium*, offering a more robust framework for species delimitation and understanding of pathogenicity [24].

The adoption of molecular tools has significantly improved the resolution of *Fusarium* species. The internal transcribed spacer (ITS) region was the first molecular marker widely used in fungal systematics. However, it has limited resolution at the species level for *Fusarium*, particularly due to its conserved nature among closely related taxa and the presence of paralogous copies complicating alignment and interpretation [25]. Studies have demonstrated that while the ITS region provides useful information for taxonomic classification, analyses of the ITS region in *Fusarium* species have shown it to be phylogenetically uninformative in some cases, resulting in misleading taxonomic assignments [26,27]. TEF1-α became the second major marker, providing greater resolution, yet still falling short when used in isolation. Although ITS and TEF1-α remain widely used markers for *Fusarium* species identification and phylogenetic analysis, their resolution is often insufficient for distinguishing closely related or cryptic species due to the genus’s high evolutionary complexity [17,28]. Also, many studies rely solely on traditional loci such as ITS and TEF1-α for the identification of *Fusarium* species, especially within species complexes [29]. This has led to recommendations for incorporating additional markers, such as RPB2 in molecular studies to achieve higher levels of precision in species identification [30]. Indeed, the combination of multiple genetic markers has been shown to significantly enhance the resolution of *Fusarium* phylogenetics, allowing for better discrimination among species and improving our understanding of their ecological and pathogenic roles [31].

Future research should adopt MLST and genome-scale data to enhance taxonomic resolution and accurately capture pathogenic and ecological diversity across *Fusarium* lineages [32,33]. To address these challenges, additional multilocus sequencing, typically using markers such as RPB1 and RPB2 and TUB2 has become a standard approach for delimiting species boundaries in the *Fusarium* genus [34]. For instance, phylogenetic analyses of RPB1 and RPB2 have resolved species within several complexes and supported the monophyly of *Fusarium* [35]. TUB2 has enhanced species delimitation in the *Fusarium* genus by providing additional phylogenetic resolution, particularly when ITS, TEF1-α, and RPB1/2 fail to discriminate closely related or cryptic taxa. Its sequence variability complements other loci in multilocus analyses, supporting more robust species boundaries, especially in complexes like *F. fujikuroi* and *F. sambucinum* [36,37]. However, challenges remain, including occasional gene tree discordance, amplification difficulties, and limited reference coverage, underscoring the need for standardized multilocus or genome-scale approaches. Frameworks such as Phylogenetic Species Recognition (PSR) and Genealogical Concordance Phylogenetic Species Recognition (GCPSR) have proven particularly effective in resolving cryptic species within major groups like FOSC and FSSC [33,38].

**Figure 1:**
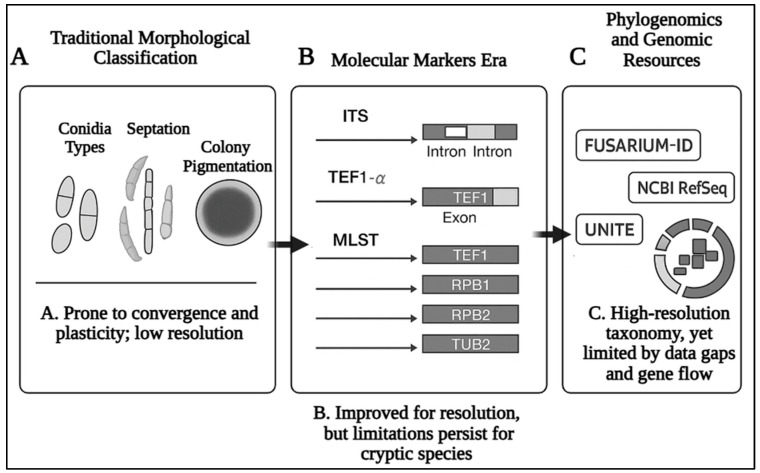
The evolution of *Fusarium* taxonomy: A comparative timeline of morphological, molecular, and genomic approaches to species delimitation. Each phase highlights advances in species resolution alongside persisting challenges such as cryptic diversity and horizontal gene transfer.

Recent advancements in next-generation sequencing (NGS) and phylogenomics have refined our understanding of *Fusarium* evolution (Figure 1). Phylogenomic analyses utilizing hundreds to thousands of single-copy orthologous genes have clarified deep phylogenetic relationships and confirmed the monophyly of the genus [39]. These studies have also highlighted genome dynamics, such as gene duplications and horizontal gene transfers, that contribute to the adaptability and pathogenicity of *Fusarium* species. For example, horizontal gene transfer has been linked to the emergence of host-specific lineages during successive outbreaks of coffee wilt disease [40]. However, despite these advances, species delimitation remains challenging due to incomplete lineage sorting, interspecific gene flow, and the limited availability of type material and genome-scale data for many underexplored taxa, particularly those associated with wild hosts or remote ecological regions [41]. Phylogenomic studies emphasize the necessity of employing comprehensive genetic datasets to capture the diversity within *Fusarium*. For instance, studies have demonstrated that the genus encompasses over 400 phylogenetically distinct species grouped into numerous complexes, such as FOSC and FSSC [28,42]. The development of curated, publicly accessible genomic repositories and barcoding databases such as FUSARIUM-ID, Fusarioid-ID, and UNITE has been instrumental in improving species resolution, facilitating accurate diagnostics, and promoting taxonomic consistency across research communities [43]. Many *Fusarium* species, particularly those from underexplored habitats or non-cultivated hosts, lack representation in reference databases, limiting the utility of barcoding tools for comprehensive identification. Additionally, discrepancies in marker choice, inconsistent taxonomic frameworks, and variable data quality across repositories can lead to misidentifications or conflicting results. Integrating multilocus and genome-scale data into these platforms, along with community-driven curation and type-material anchoring, will be essential for ensuring taxonomic reliability and expanding coverage of the full phylogenetic and ecological diversity within the *Fusarium* and Fusarioid clades.

### 2.2. Rise and Relevance of Fusarium Species Complexes: Evolutionary Divergence, Taxonomic Challenges, Intraspecific Variability and Pathogenic Implications

The rise in species complexes represents a significant aspect of evolutionary biology and taxonomy, particularly regarding the genus *Fusarium*, which exemplifies the complexities caused by ecological variations and genetic divergence [22,44]. A species complex refers to a group of closely related species that are morphologically similar but genetically distinct, resulting in challenges in identification and treatment, particularly in agriculture. These species complexes have surfaced as significant entities in the taxonomy of *Fusarium* [45]. *Fusarium fujikuroi* (FFSC), FGSC, *Fusarium incarnatum-equiseti* (FIESC), FOSC, FSSC, and *Fusarium tricinctum* (FTSC), species complexes are prominent examples, with each complex highlighting unique ecological niches and pathogenic potential [46]. Molecular systematics has revealed that many named species are in fact species of complex groups of genetically distinct but morphologically indistinguishable taxa, which has revolutionized our understanding of *Fusarium* evolution and pathogenicity [3].

**Figure 2:**
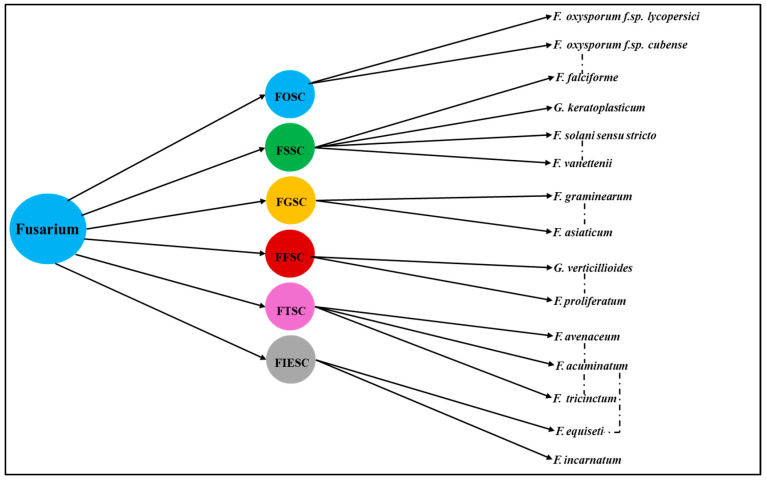
Phylogeny relationships and pathogenic overlap among *Fusarium* species complexes. Dashed arrows indicate reported overlaps in crop host range, morphological features, or disease symptoms between species from different complexes. FOSC—*Fusarium oxysporum* species complex. FSSC—*Fusarium solani* species complex. FGSC—*Fusarium graminearum* species complex. FFSC—*Fusarium fujikuroi* species complex. FTSC—*Fusarium tricinctum* species complex. FIESC—*Fusarium incarnatum-equiseti* species complex.

These *Fusarium* species complexes vary in host specificity, pathogenicity, and toxin production [3]. The morphological overlap among *Fusarium* species from different complexes frequently leads to misidentification and complicates disease diagnosis in crops like maize, tomato, rice, and cereals [47,48] (Figure 2). For instance, vascular wilt symptoms in tomato can be caused by both *F. oxysporum* and *F. solani* (wilting occurring in rare instances as a secondary symptom), while maize stalk rot may be attributed to *F. verticillioides* (FFSC) or co-occurring *F. solani* strains [49,50] and has accelerated the shift toward multilocus sequence typing and genome-informed phylogenetics as standard practice. Emerging research reveals that species within the *Fusarium* complex also exhibit extensive genetic and phenotypic variation in plant hosts, which plays a critical role in their virulence, host range, and evasion of plant defense responses [51,52,53]. For example, members of FOSC can display varying degrees of aggressiveness and host specificity, often mediated by lineage-specific chromosomes encoding effector proteins that suppress plant immunity [13]. Similarly, strains within FSSC have demonstrated the ability to colonize diverse plant tissues, aided by enzymes that degrade cell walls and subvert plant signaling pathways [14,54]. These adaptive mechanisms not only facilitate cross-species infection but also complicate disease management strategies, emphasizing the ecological and agricultural significance of *Fusarium* diversity and its evolutionary capacity to overcome plant defenses.

Moreover, taxonomic classification has undergone significant refinement within the *Fusarium* genus, particularly concerning species complexes that challenge traditional morphological and pathogenicity-based frameworks. A prime example is FSSC, where historical use of the *forma specialis* designation, such as in *F. solani* f. sp. *pisi* has proven inadequate for reflecting phylogenetic diversity and host range specificity [55]. Molecular phylogenetics, especially MLST and whole-genome analyses, have revealed that what was once considered a single species based on pathogenic behavior encompasses numerous cryptic taxa with distinct evolutionary lineages and ecological adaptations [22,56], particularly given the agricultural relevance of FSSC members [57]. In addition to interspecific divergence, intraspecific variability, such as the occurrence of physiological races within *formae specialis*, adds further complexity. For example, *F. oxysporum* f. sp. *lycopersici* comprises three main races (1, 2, and 3), each defined by its ability to overcome specific tomato resistance genes [58]. A study from Baja California, Mexico, identified multiple races co-occurring within tomato fields, underscoring the ongoing diversification and adaptation of this pathogen under selective host pressure [59]. Similarly, *F. oxysporum* f. sp. *melonis*, affecting cucurbits, exhibits a well-defined race structure in Italy, where isolates belonging to races 1 and 2 were found to differ significantly in pathogenicity and genetic makeup, revealing strong geographic and host-specific adaptation [60]. These race dynamics reflect host–pathogen coevolution and present major challenges for resistance breeding, epidemiological surveillance, and effective disease control. Recent studies emphasize the necessity of abandoning the *formae specialis* nomenclature in favor of phylogenetically informed classifications, which not only improve species resolution but also enhance our understanding of host–pathogen interactions and cross-kingdom pathogenicity [55,57]. Integrative taxonomy combining morphological traits, pathogenicity assays, and genomic data is now indispensable for accurate *Fusarium* species identification. The study of species complexes is further underscored by broader implications in ecology and evolutionary biology. Alterations in species distributions, as witnessed in invasive organisms, indicate niche shifts due to environmental changes, which also applies to *Fusarium* species and native species adapting to new ecological contexts. This dynamic illustrates how ecological pressures influence species complex evolution [3,5]. The understanding of these processes not only informs taxonomy but also aids in conservation efforts by delineating species that might otherwise be overlooked, especially in environments facing multiple stressors. In summary, the rise in species complexes such as those represented by the genus *Fusarium* emphasizes the complex interplay of genetics, ecology, evolutionary processes, and recognizing ecological roles and resistance mechanisms are pivotal for advancing our understanding and management of these biologically significant groups.

### 2.3. The Neocosmospora Debate: Taxonomy Reshuffled, or Genus Redefined?

The taxonomic classification of FSSC remains a subject of considerable debate in agricultural mycology, particularly concerning its proposed reclassification into the genus *Neocosmospora*. This shift has been advocated based on multilocus phylogenetic analyses, with proponents arguing that it reflects a more precise evolutionary understanding [41]. However, critics suggest that such a segregation could diminish diagnostic clarity and taxonomic stability, which are pivotal in managing *Fusarium* infections [61]. Phylogenetic studies using multilocus sequence data and genome-scale analyses have consistently demonstrated that members of the FSSC, including *F. solani sensu stricto*, form a distinct monophyletic clade that is evolutionarily divergent from the core *Fusarium* lineage [32]. In response, some taxonomists have proposed reclassifying these organisms under *Neocosmospora*, a move that seeks to align nomenclature with phylogenetic evidence [62]. However, in agricultural contexts, this proposed separation poses significant challenges. *Neocosmospora solani*, which includes strains formerly identified as *F. solani*, causing diseases in legumes, cucurbits, and solanaceous crops, is one of the most widely distributed and economically impactful plant pathogens worldwide [46]. *Fusarium* carries historical and regulatory significance, and abrupt taxonomic changes without broad community consensus can hinder communication and the practical application of agricultural research findings.

Moreover, the significance of FSSC in agriculture is substantial, as members of this complex are major pathogens responsible for diverse plant diseases across a wide range of economically important crops. The FSSC includes highly virulent strains that cause root rot, stem rot, and seedling blight, leading to considerable yield losses and affecting plant health in both field and greenhouse conditions [61]. The taxonomic complexity of the FSSC, compounded by its morphological similarity to other *Fusarium* species, poses challenges for accurate identification and effective disease management. Molecular typing has become essential to resolve these ambiguities and ensure precise diagnostics in phytopathological studies. Recent phylogenetic analyses support the segregation of certain FSSC members into the genus *Neocosmospora*, based on distinct genetic and morphological characteristics. However, this taxonomic revision remains controversial, particularly due to the long-standing agronomic relevance of the FSSC within the broader *Fusarium* genus and concerns over continuity in disease monitoring and control strategies [31,61].

Recent phylogenomic investigations have provided compelling evidence that FSSC shares a core evolutionary lineage with other major *Fusarium* clades, supporting its continued placement within the genus. Whole-genome analyses have revealed substantial genomic synteny and conserved orthologous gene content across FSSC and other *Fusarium* lineages, suggesting that FSSC members, including agriculturally significant pathogens of legumes and solanaceous crops, are not sufficiently divergent to justify generic separation [63]. These genomic similarities underscore a broader shift in fungal systematics toward phylogenomically informed classification schemes that prioritize shared evolutionary ancestry over ecological niche or morphological traits [64,65]. This unified taxonomic framework is particularly important in agricultural contexts, where precision in pathogen identification directly impacts disease surveillance, resistance breeding, and biosecurity strategies. For instance, the retention of FSSC in *Fusarium* facilitates the use of standardized molecular barcoding tools (e.g., TEF1-α and RPB2 loci) that underpin many diagnostic platforms in agricultural research and regulatory systems [66]. In summary, while phylogenomic evidence supports a move towards recognizing *Neocosmospora* as a distinct entity, the practical implications for diagnostics and the continuity of historical nomenclature raise critical questions about the stability and operational utility of such taxonomic changes. Additionally, whereas monophyly is necessary, it is not sufficient for genus-level classification [24] but rather an integrated approach combining morphology, ecology, biochemistry and phylogeny. The ongoing debate reflects broader issues within fungal systematics, where phylogenetic integrity must be balanced against practical considerations in agricultural settings.

### 2.4. Emerging Fusarium Pathogens in Niche Crops and Geographies

The landscape of *Fusarium*-related plant diseases is rapidly shifting, with both novel and previously known species emerging in unexpected host crops and regions, driven by ecological, agricultural, and climate dynamics (Table 1). *Fusarium zanthoxyli* was recently identified as the causal agent of stem canker in *Zanthoxylum bungeanum* (Sichuan pepper), a high-value spice crop widely cultivated in northern China [67,68]. This pathogen invades woody tissues, resulting in cankers, dieback, and often tree mortality, causing substantial yield and economic losses. Phylogenetic analyses position *F. zanthoxyli* within the newly defined *Fusarium torreyae* species complex (*FTOSC*), which also includes *F. torreyae*, a pathogen known to devastate *Torreya taxifolia* populations in North America [69]. Notably, the FTOSC represents a group of wood-adapted *Fusarium* pathogens that diverged significantly from typical herbaceous-host lineages. Comparative genomic studies estimate the divergence of *F. zanthoxyli* from other *Fusarium* species occurred between 17.2 and 27.5 million years ago and uncovered 137 lineage-specific effector proteins that likely contribute to host specificity and virulence in woody plants [70]. This molecular specialization underscores the evolutionary plasticity of *Fusarium* in colonizing novel ecological niches. Similar adaptability has been observed in well-known pathogens such as *F. oxysporum* f. sp. *cubense* Tropical Race 4 (Foc TR4), the causal agent of banana wilt, which has expanded rapidly from Southeast Asia to the Middle East, Africa, and most recently Latin America, threatening global banana production [71,72,73]. The emergence of *F. zanthoxyli* in perennial spice systems, alongside the re-emergence of virulent *F. oxysporum* strains in globally strategic crops like banana, highlights the urgency of targeted pathogen surveillance and the development of integrated, crop- and region-specific disease management strategies.

FIESC and FFSC, long associated with cereals, are now emerging in diverse agroecosystems, including medicinal herbs, horticultural crops, and legumes across Asia, Africa, and South America [74,75]. In China, Wang et al. [76] identified nine novel FIESC species from a variety of hosts, underscoring the taxonomic complexity and diagnostic challenges within this group. These species exhibited diverse virulence profiles and mycotoxin production capabilities, which complicate risk assessments in traditional and alternative cropping systems. In Brazil and other parts of South America, FIESC members have been implicated in diseases of rice and soybean, with some isolates producing significant levels of zearalenone and deoxynivalenol [77,78,79]. Similar trends are emerging in African countries, where FIESC strains have been isolated from legumes and horticultural crops, raising concerns over regional food safety and market access. These developments stress the need for region-specific diagnostic tools and comprehensive toxigenic profiling to inform appropriate disease control strategies.

FTSC is also gaining prominence as a pathogen group affecting niche and high-value crops. Members such as *F. acuminatum*, *F. avenaceum*, and *F. tricinctum* have been increasingly reported from soybean [80], medicinal herbs [37,81,82,83], cereals [84], and temperate fruits, including apples, strawberries, and raspberries, across Asia and Europe. *F. avenaceum* is particularly concerning due to its cross-pathogenic behavior; it has been linked to root rot in pulses and cereals [7] and raspberry [85]. Moreover, FTSC members are increasingly associated with storage rots in postharvest fruit systems, reducing shelf life and marketability. The frequent isolation of FTSC species from geographically and taxonomically diverse hosts suggests a broad ecological amplitude and underscores the importance of enhanced molecular diagnostics, regionally tailored management practices, and cross-border phytosanitary coordination. Collectively, these findings illustrate how emerging *Fusarium* pathogens are reshaping the disease landscape in non-cereal crops, with implications for food security, biodiversity, and international trade. Their rising prevalence is closely linked to agricultural intensification, climate variability, global seed exchange, and expanding monocultures. A proactive and globally coordinated response integrating pathogen genomics, surveillance, and crop-specific disease management is critical to mitigate the expanding threat of *Fusarium* in niche and non-traditional agroecosystems.

**Table 1:** Geographic distribution and host range of emerging *Fusarium* pathogens in niche and high-value crops.

*Fusarium* Species	Species Complex	Primary Host(s)	Crop Type	Region	References
*F. zanthoxyli*	FTOSC	*Zanthoxylum bungeanum*	Spice crop (woody)	Northern China	[67,68]
*F. torreyae*	FTOSC	*Torreya taxifolia*	Tree (conifer)	North America	[69]
*F. oxysporum* f. sp. *cubense TR4*	FOSC	Banana (*Musa* spp.)	Fruit crop (perennial herb)	Southeast Asia, Middle East, Africa, Latin America	[71,72,73]
Novel FIESC spp.	FIESC	Various herbs and crops	Medicinal, legumes	China	[76]
FIESC spp.	FIESC	Rice, soybean, legumes	Cereals, legumes	South America, Africa, Europe	[77,79,80]
*F. acuminatum*, *F. avenaceum*, *F. tricinctum*	FTSC	Soybean, medicinal herbs, fruits, cereals	Multiple	Asia, Europe	[80,82,86]
*F. avenaceum*	FTSC	Raspberry, pulses, cereals, soybean	Berries, legumes	Europe, North-Estern China	[7,80,85,87]

### 2.5. Phylogenomics of Fusarium: Insights from Whole-Genome Data

Whole-genome sequencing (WGS) has dramatically revolutionized the understanding of evolutionary complexity in the *Fusarium* genus. Among the most remarkable features is the diversity of genome size and GC-content, particularly within the FFSC and the FIESC [46,88]. For example, species from FFSC present genome sizes varying from 39 to 56 Mb and strong heterogeneity in the GC content, indicating complex evolutionary story and lineage-specific genome plasticity [89,90]. Similarly, an assembly of *F. equiseti* genomes from multiple agroecosystems showed the presence of numerous variabilities in repetitive DNA and mobile elements, which probably leads to the genomic heterogeneity [91]. The genome of *F. solani* f. sp. *melongenae* with unique ankyrin-repeat proteins exhibited large-scale structural changes including inversions and rearrangements [92]. These changes in genome architecture could contribute to ecological specialization and host range broadening. This flexibility indicates that *Fusarium* species have developed separate genome architectures, which have allowed adaptation to selective pressures, which is supported by the observed genetic diversity and haplotype variation within *Fusarium* isolates. Yet understanding why this variability occurs is a key problem, since it requires understanding the pressures which exert their influence at the level of size and genome organization.

Comparison of core proteome also revealed a conservation profile among the *Fusarium* species; indicative of fundamental cellular processes and functions shared among species that reside in different members in the genus [31,93]. For instance, FGSCs encoding biosynthesis pathways of secondary metabolites that are important to these fungi for ecological fitness are observed to be conserved in various *Fusarium* species, suggesting the same evolutionary origin [94]. Nevertheless, an overall high degree of conservation in the core proteome in the genus is maintained, in which *Fusarium* spp. harbor a conserved core genome of hundreds of single gene orthologs shared within pathogenic and non-pathogenic strains, suggesting that it is evolutionarily stable [63,95]. These conserved proteins are often involved in basal cellular processes, including those of primary metabolism, DNA replication and translation. The conservation of these sequences renders them suitable targets for species specific identification and robust phylogenetic inference. Moreover, this evolutionary conservation provides a molecular framework for taxonomic stability, allowing for high-resolution phylogenomic trees that are representative of species relationships [96]. The problem is the reliable resolution of these crosstalk effects into conserved gene interaction networks with environmental variables and, especially, how these play a role in species divergence and adaptation [97,98], which may be indispensable for implementation of standardized molecular barcoding sets and next-generation diagnostic tools for crop protection.

In contrast to the conserved core, the genomic structure in *Fusarium* is thus an example of functional diversification also for accessory chromosomes (ACs) which are of particular interest in the FOSC and FFSC where ACs are required for increased pathogenicity and specialization towards certain hosts by carrying genes that are responsible for these beneficial characteristics [99,100]. Functional differentiation is focused in the ACs, especially in the FOSC and FSSC. These ACs contain genes involved in host–pathogen interactions, including virulence effectors secreted in xylem proteins (SIX effectors) and specialized secondary metabolite clusters [53]. ACs have been reported to have extensive structural variation and can be often horizontally transferred among strains, providing a potential reservoir for transfer of pathogenic evolution [101,102]. Most *Fusarium* genomes have large accessory regions (i.e., gene-based regions present in only some strains of a species) that can significantly differ in content, reflecting their potential as contributors to host-specific interactions. It has also been demonstrated that these accessory elements can be gained or lost through horizontal gene transfer, adding complexity to clade-specific evolution of genes in *Fusarium* [99]. Phytopathogenic *Fusarium* species have been associated with host specificity jumps in pulse crops and some medicinal plants, and to a lesser extent in pathogens of xenobiotic degrading plant-pathogens [7,82]. Yet, the mechanisms by which accessory chromosomes shape pathogenicity and environmental response are still not fully understood. These findings highlight the plasticity of the *Fusarium* genome and its ability to colonize new environments.

A further insight that can be gleaned from genome-wide data is the extent of gene duplication in lineage-specific regions, which is commonly associated with host adaptation and the evolution of secondary metabolism. Similarly, gene duplications related to metabolic pathways that result in variation in metabolism (mycotoxins production) imply adaptive responses to environmental stresses or host interactions [81,97]. There is evidence that some duplications of individual gene clusters, but not class of gene clusters, such as the PKSs, NRPSs, and P450s, are associated with the ability of various *Fusarium* strains to produce specific mycotoxins or other types of secondary metabolites [103]. These genomic adaptations improve the fitness and competitive ability of the *Fusarium* species on unique hosts and subvert disease diagnostics and toxin surveillance. However, more work is needed to fully elucidate the long-term evolutionary dynamics of these gene duplications and the effects on pathogen virulence and resistance. Linking genetic adaptations with functional effects across different ecological circumstances is an ongoing challenge.

## 3. Genomic Insights and Functional Genomics: Exploring Recent Genomic Studies and Their Implications for Understanding Pathogenicity and Resistance Mechanisms

Recent advances in genomic research have deepened our understanding of *Fusarium* species, shedding light on their evolution, pathogenicity, and adaptive mechanisms [31,53,104]. High-quality genome assemblies and annotations have revealed mobile pathogenicity chromosomes, emphasizing the role of horizontal gene transfer in shaping virulence traits [13,105,106,107]. Comparative genomics has identified key genes implicated in infection processes, host adaptation, and secondary metabolism clusters that influence toxin production and environmental interactions [108,109,110,111]. Functional genomics tools such as CRISPR/Cas9 and RNA-Seq continue to refine gene function studies, enabling precise manipulation of *Fusarium* genomes and facilitating investigations into molecular mechanisms governing fungal pathogenicity [112,113,114]. Phylogenomic analyses provide critical insights into species relationships within *Fusarium* complexes, highlighting genetic diversity and evolutionary trajectories across different lineages [96]. Together, these advances contribute to a more comprehensive framework for understanding *Fusarium* biology, with implications for disease management and agricultural sustainability.

### 3.1. Genomic Architecture of Fusarium Species

Recent genome assemblies and annotations of various *Fusarium* species, such as *F. graminearum*, *F. verticillioides*, and *F. oxysporum*, have revealed complex genomic architectures comprising both core and accessory chromosomes [104]. Core chromosomes encode genes essential for basic cellular functions, while accessory chromosomes are enriched with genes involved in pathogenicity, environmental adaptation, and secondary metabolism. One of the most striking discoveries in *F. oxysporum* is the presence of mobile pathogenicity chromosomes, which confer host-specific virulence and can be horizontally transferred between strains [13]. This process, known as Horizontal Chromosome Transfer (HCT), has been most extensively studied in *F. oxysporum*, but emerging evidence suggests that HCT may also occur in other *Fusarium* species, potentially contributing to the rapid evolution of pathogenic traits across the genus. Here are some examples and key findings related to HCT in different *Fusarium* species:

*F. oxysporum*: HCT has been extensively studied in *F. oxysporum*, particularly in relation to pathogenicity chromosomes encoding host-specific virulence factors. These transfers contribute to the emergence of new races/pathotypes and *formae speciales* within *F. oxysporum*. A prominent example is the SIX (Secreted In Xylem) gene family, located on these accessory chromosomes, which is involved in virulence and can be horizontally transferred [105]. Comparative genomics and pathogenicity phenotyping have been used to explore the role of HCT in the evolution of *F. oxysporum* f. sp. *fragariae*, the causative agent of Fusarium wilt in strawberries [106]. Isolates from four continents revealed two distinct pathogenicity syndromes: one characterized by chlorosis (yellows-fragariae) and the other by wilting (wilt-fragariae). Notably, all yellows-fragariae isolates carried a pathogenicity chromosome, chrY-frag, which was horizontally transferred at least four times. This chromosome was linked to virulence on specific cultivars and encoded predicted effectors that were significantly upregulated during infection. The absence of chrY-frag in wilt-fragariae isolates indicated that pathogenicity could evolve independently in different strains. Furthermore, interactions between *F. oxysporum* f. sp. *apii* race 4 and *F. oxysporum* f. sp. *coriandri* were also studied, showing that under conditions conducive to somatic compatibility or conidial anastomosis tube formation, *F. oxysporum* species can perform nuclear transfer and HCT, leading to the development of more virulent genotypes [107].

Beyond *F. oxysporum*, several other *Fusarium* species exhibit genomic traits suggestive of HCT involvement. In *F. graminearum*, there is emerging evidence that gene clusters involved in secondary metabolite production, such as those for trichothecene biosynthesis (e.g., deoxynivalenol, DON), may have been horizontally transferred between *F. graminearum* and related species. Such transfers could enhance pathogenicity and adaptability to cereal crops, although more definitive evidence is still needed [104]. In *F. verticillioides*, the causal agent of ear rot in maize, variability in fumonisin biosynthetic gene clusters among different strains also suggests possible involvement of HCT in shaping strain-specific pathogenic traits and environmental adaptation [108]. Likewise, in *F. solani*, genomic analyses have revealed lineage-specific genes likely acquired through HCT that may contribute to its opportunistic pathogenicity and ability to thrive in diverse ecological niches [104]. *Fusarium fujikuroi*, known for producing gibberellins and other secondary metabolites, also harbors diverse biosynthetic gene clusters with regional variation, potentially due to HCT. These clusters are linked to pathogenicity and host specialization, enhancing the species’ metabolic flexibility and environmental adaptability [109]. While *F. oxysporum* is the best-studied species for HCT, emerging research suggests that other *Fusarium* species may also utilize HCT to enhance pathogenicity, expand host range, and adapt to different environmental conditions. Horizontal transfer of chromosomal regions involved in virulence factors and secondary metabolite production could be a key mechanism driving the emergence of new pathogenic races and increased virulence in *Fusarium* species. However, more genomic studies and pathogenicity phenotyping are needed to validate these findings across various *Fusarium* species.

### 3.2. Secondary Metabolites, Genome Plasticity, and Host Specialization

Secondary metabolites and genome plasticity are key drivers of pathogenicity, host specialization, and environmental adaptation in *Fusarium* species. Economically important species such as *F. proliferatum* and *F. fujikuroi* harbor extensive biosynthetic gene clusters responsible for the production of secondary metabolites including beauvericin, fumonisins, moniliformin, gibberellins, and fusarin C. These mycotoxins not only contribute to virulence and host colonization but also enhance the pathogen’s competitive fitness in planta. Comparative genomic analyses of *F. fujikuroi* have revealed a significantly expanded repertoire of polyketide synthase (PKS) and nonribosomal peptide synthetase (NRPS) genes relative to non-pathogenic species, underscoring the role of secondary metabolism in the evolution of a pathogenic lifestyle [109].

Furthermore, isolates of *F. fujikuroi* from different geographical regions display substantial variation in the presence and expression of secondary metabolite gene clusters. This leads to distinct mycotoxin profiles, which correlate with differences in virulence, host range, and ecological adaptation. The presence of lineage-specific clusters, such as those involved in gibberellin and fusarin C biosynthesis, enhances metabolic plasticity and facilitates adaptation to diverse host environments [109].

Complementing the role of secondary metabolites, genome plasticity—particularly the presence of accessory genomic elements—further drives host specialization in *Fusarium* species. Pan-genomic analyses reveal that approximately 20–30% of the *Fusarium* genome consists of accessory genes that are highly variable among isolates [13,109,110,111]. These accessory regions are enriched in genes related to pathogenicity, secondary metabolism, and environmental sensing, providing a flexible genomic toolkit for rapid adaptation to novel hosts or fluctuating environmental conditions.

Together, the dynamic interplay between secondary metabolite diversity and genome plasticity defines the remarkable ecological adaptability and host specificity of *Fusarium* pathogens, positioning them as versatile and persistent threats in agricultural systems.

#### 3.2.1. Functional Genomics and Pathogenicity Mechanisms

Recent advancements in functional genomics have significantly enhanced our understanding of the pathogenicity mechanisms in *Fusarium* species. Tools such as CRISPR/Cas9 and RNA-Seq have revolutionized our ability to dissect the molecular basis of fungal virulence, providing insights into the roles of key genes involved in infection and host interaction.

The CRISPR (clustered regularly interspaced short palindromic repeats)-Cas9 system, derived from the bacterial and archaeal immune system, has been developed into a powerful gene editing tool widely applied to *Fusarium* species to elucidate the functions of specific genes related to pathogenicity. This technology has allowed for the targeted knockout of genes thought to be essential for virulence [112,113,114]. For example, in *F. oxysporum*, the FTF (Fusarium transcription factor) gene family, consisting of the conserved FTF2 and multiple FTF1 copies (exclusive to *F. oxysporum*), plays a key role in pathogenicity regulation. Functional studies using RNA interference demonstrated that knockdown of FTF1 and FTF2 led to a strong reduction in virulence, correlated with decreased expression of SIX effector genes and their regulator SGE1, suggesting that FTF1 paralogs control the activation of effector gene expression critical for host infection [115,116].

Mutants lacking FTF1 showed a markedly reduced ability to cause disease, highlighting the key role of this transcription factor in regulating both effector gene expression and secondary metabolism pathways associated with virulence in *F. oxysporum*. In parallel, the TRI gene cluster, responsible for trichothecene biosynthesis in *F. graminearum*, is critical for pathogenicity. Disruption of key TRI genes, such as TRI5 or TRI14, significantly impairs fungal spread and DON accumulation in wheat, confirming that trichothecenes function as essential virulence factors [13,117].

In addition to genes encoding secondary metabolites, CRISPR/Cas9 has also been employed to investigate other virulence-related genes, such as those involved in cell wall integrity and host recognition. In *F. oxysporum*, the Slp1 gene, which encodes a secreted LysM protein that suppresses plant immune responses, has been identified as essential for host-specific pathogenicity, as its disruption leads to loss of virulence on certain plant hosts [118]. Given its critical role, Slp1 represents a promising target for functional knockout using CRISPR/Cas9 technology, which could validate and exploit its role in virulence suppression for disease control strategies. These findings highlight the capacity of CRISPR/Cas9 for functional validation of genes involved in *Fusarium* pathogenicity and host specialization.

RNA-Seq technology has become a cornerstone in functional genomics, offering high-resolution insights into transcriptional reprogramming during plant-pathogen and plant-beneficial microbe interactions. In *Fusarium* species, RNA-Seq has enabled the identification of host-specific expression patterns of virulence-associated genes, including those encoding effector proteins and cell wall-degrading enzymes (CWDEs), which are essential for successful colonization and infection. For example, in *F. oxysporum* f. sp. *fragariae*, RNA-Seq analyses revealed that specific effector genes were significantly upregulated during the infection of susceptible strawberry cultivars, highlighting their role in host adaptation and pathogenicity [119]. Additionally, genes encoding CWDEs, such as cellulases, pectinases, xylanases, and glucanases, have been consistently identified as major factors facilitating plant tissue invasion by degrading structural barriers and suppressing host defense responses.

Similarly, in *F. graminearum*, which causes Fusarium head blight in cereals, transcriptomic analyses during wheat and maize infection have demonstrated the simultaneous upregulation of TRI genes responsible for trichothecene biosynthesis and multiple genes coding for CWDEs. These coordinated transcriptional responses reflect a multifaceted infection strategy aimed at both chemical and enzymatic suppression of plant immunity [120].

Importantly, RNA-Seq has also been instrumental in characterizing beneficial microbes such as *Trichoderma* spp., which are widely employed as biocontrol agents. A recent comparative transcriptomic study focused on two *T. afroharzianum* isolates (Th19A and Th4) during their interaction with *F. virguliforme*, the soybean sudden death syndrome pathogen. Despite being of the same species, these isolates exhibited markedly different antagonistic behaviors—Th19A overgrew the pathogen, whereas Th4 induced a clear inhibition zone. These phenotypic differences were reflected at the transcriptomic level, revealing significant changes in the expression of genes encoding secreted proteins, including CAZymes and CBM1-domain-containing proteins, in both *Trichoderma* and *F. virguliforme*. Notably, some of these genes were upregulated even before physical contact occurred, suggesting that volatile-mediated recognition may play a role in early signaling events during biocontrol interactions [121].

Collectively, these studies underscore the versatility of RNA-Seq as a tool for unraveling the complex molecular interactions between plants, pathogens, and beneficial microbes. Insights gained from such analyses are critical for the development of targeted and efficient biological control strategies, tailored to specific host–pathogen contexts and microbial isolates.

In addition to genome editing and transcriptomic approaches, the identification of key transcription factors has been pivotal for understanding how *Fusarium* species regulate host-specific pathogenicity and secondary metabolism. Table 2 summarizes major transcription factors known to control pathogenicity genes and the synthesis of toxic secondary metabolites in various *Fusarium* species.

#### 3.2.2. Proteomics and Secreted Virulence Factors

Proteomic studies complement transcriptomic data by identifying secreted virulence factors such as hydrolases, lipases, and necrosis-inducing proteins, which play critical roles in pathogenicity. Secreted proteins, including hydrolases, lipases, and necrosis-inducing proteins, have been detected in the secretomes of various *Fusarium* species, emphasizing their involvement in tissue colonization and nutrient acquisition. In *F. oxysporum*, a necrosis- and ethylene-inducing peptide (Nep1)-like protein was found to trigger programmed cell death in *Arabidopsis thaliana*, creating an environment conducive to fungal growth and colonization [130].

Similarly, *F. verticillioides*, a maize pathogen, secretes a variety of proteins, including lipases and cutinases, which are involved in breaking down plant cuticle and cell wall components, facilitating fungal entry and colonization [131]. These secreted proteins not only contribute to virulence but also help the fungi obtain nutrients from the host, a critical step in its survival and pathogenicity.

Proteomics has also led to the identification of a range of effector proteins that help the pathogen evade the plant immune system. For example, *F. oxysporum* secretes effectors known as SIX proteins, which are highly conserved and have been shown to play essential roles in promoting virulence in different plant hosts. One such effector, SIX6, contributes to virulence and is capable of suppressing host defense responses, including I-2-mediated cell death in tomato [132]. These effectors manipulate plant immune responses, enabling the pathogen to suppress host defenses and establish successful infections.

### 3.3. Resistance Mechanisms in Host Plants

Plant defense against *Fusarium* involves the recognition of pathogen-derived molecules and the activation of immune responses. These defenses include both basal immunities, triggered by conserved microbial signatures, and specific resistance mediated by resistance (R) genes that recognize pathogen effectors. Advances in genome-wide association studies (GWAS), gene expression profiling, and functional genomics have facilitated the identification of candidate resistance genes across a wide range of crop species. These discoveries have deepened our understanding of host–pathogen interactions and have significantly supported breeding programs aimed at developing *Fusarium*-resistant cultivars. Traditional breeding methods, such as the selection of quantitative trait loci (QTLs) and R genes, have historically provided durable and broad-spectrum resistance by leveraging naturally occurring genetic diversity within crop germplasm. While effective, these approaches are often time-consuming and limited by the genetic background of available cultivars. In contrast, modern biotechnological tools such as CRISPR/Cas9 genome editing and RNA interference (RNAi) allow for precise and targeted manipulation of host genes involved in immunity, accelerating the development of resistant varieties. CRISPR can be used to knock out or modify susceptibility genes and to activate defense-related genes. RNAi, particularly through host-induced gene silencing (HIGS), enables the suppression of key fungal pathogenicity genes. Despite their potential, these technologies face challenges related to regulation, biosafety, and public acceptance in many countries. They also require well-established transformation systems, which are not available for all crops. For this reason, combining traditional breeding approaches with modern molecular tools offers a promising strategy to accelerate resistance development against *Fusarium* pathogens. Table 3 provides a summary of economically important *Fusarium* species, their host crops, and associated resistance genes.

### 3.4. Phylogenomics and Comparative Genome Analysis Across Species Complexes

Phylogenomic approaches integrate evolutionary and functional data to resolve relationships within species complexes like *F. oxysporum* species complex (FOSC) and *F. fujikuroi* species complex (FFSC). Comparative analyses across these complexes reveal that horizontal gene transfer, gene duplication, and differential loss shape the evolution of pathogenicity traits [13]. For example, the divergence of *formae speciales* within *F. oxysporum* is linked to acquisition and diversification of pathogenicity chromosomes, with effectors evolving under strong positive selection pressures. Comparative studies also show that some secondary metabolite clusters are conserved across species, while others are uniquely expanded, offering a window into how host-specificity and virulence evolve. Pan-genomic approaches are now being used to capture the full spectrum of genetic diversity in *Fusarium*, revealing a “flexible genome” architecture that underpins both saprophytic and pathogenic lifestyles [111].

In conclusion, the integration of genomics, pathogenomics, and functional studies is reshaping our understanding of *Fusarium* pathogenicity and host resistance. This knowledge is being applied to breed resistant cultivars through marker-assisted selection (MAS), genomic selection, and genome editing. CRISPR/Cas9, RNA interference (RNAi), and new systems biology tools are poised to accelerate functional validation of resistance and virulence genes. Future work will focus on real-time pathogen monitoring, deeper understanding of epigenetic regulation of pathogenicity, and synthetic biology approaches to create durable resistance in crops. Continued efforts in multi-omics integration, real-time pathogen monitoring, and functional validation of candidate genes will be essential in managing *Fusarium*-related crop losses in a changing climate.

## 4. Pathogenicity and Host Interactions

*Fusarium* species exhibit a wide range of pathogenic behaviors, influencing agricultural ecosystems through host-specific interactions and adaptive infection strategies [9]. The pathogenic potential of *Fusarium* spp. is shaped by diverse virulence factors, allowing them to infect numerous plant species and cause devastating diseases. Host-specificity plays a crucial role in infection dynamics, with certain *Fusarium* species forming distinct *formae speciales* that target specific plant hosts. This specialization highlights the genetic and molecular adaptations that drive host–pathogen compatibility. Plants, however, are not passive victims; they deploy complex immune responses, including structural defenses and biochemical signaling, to resist Fusarium infections. In response, *Fusarium* species have evolved sophisticated counter-defense mechanisms, such as effector proteins and toxin production, to suppress host immunity and enhance disease progression [13,132]. Understanding these interactions at the molecular and genomic levels is essential for developing effective disease management strategies and mitigating the impact of Fusarium-induced crop losses.

### 4.1. Fusarium Pathogenicity

This section seeks to emphasize recent studies in understanding the pathogenicity of key *Fusarium* species and their dynamic interactions with hosts. The pathogenicity of *Fusarium* species is driven by a complex interplay of virulence factors, including effector proteins, mycotoxins, and cell wall-degrading enzymes, which collectively facilitate host colonization and disease progression [9]. Advances in genomic and molecular studies have shed light on how these pathogens adapt to diverse hosts and environmental conditions, often through horizontal gene transfer or genomic rearrangements [53]. Unraveling these adaptive strategies is critical for developing targeted interventions, such as resistant crop varieties or antifungal agents, to mitigate the devastating impacts of Fusarium-associated diseases on agriculture and food security. A prominent example of these virulence mechanisms can be found in *F. oxysporum*, which employs specialized effect proteins to manipulate host defenses and facilitate infection.

*F. oxysporum* secretes Secreted in Xylem (SIX) proteins, which suppress host immunity and facilitate colonization. For example, *F. oxysporum* f. sp. *lycopersici* (Fol) produces 14 SIX proteins, including SIX1–SIX14, with SIX4 (Avr1), SIX3 (Avr2), and SIX1 (Avr3) acting as avirulence effectors recognized by resistant tomato cultivars [13,144]. In *F. oxysporum* f. sp. *vasinfectum* (Fov), 19 FovSIX proteins were identified, with FovSIX16 experimentally confirmed as essential for virulence in cotton [144]. These effectors are often located on lineage-specific (LS) chromosomes, which can be horizontally transferred between strains, altering host-specificity [144,145].

Additionally, other *Fusarium* species produce mycotoxins (e.g., trichothecenes, fumonisins) that weaken host defenses and contaminate crops. *Fusarium graminearum* synthesizes deoxynivalenol (DON), which disrupts plant cell functions and promotes head blight in cereals [146,147]. *F. oxysporum* produces fusaric acid, a phytotoxin that induces wilting by disrupting mitochondrial function and suppressing host immune responses [148]. Secondary metabolite gene clusters (e.g., nonribosomal peptide synthetases, polyketide synthases) are enriched in LS chromosomes, linking them to pathogenicity [148].

*Fusarium* employs Cell Wall-Degrading Enzymes (CWDEs) such as pectinases, cellulases, and xylanases to breach plant cell walls. Transcriptomic studies of *F. oxysporum* f. sp. *cucumerinum* (Foc) revealed upregulation of CWDE genes during infection, enabling root colonization [149]. These enzymes are critical for nutrient acquisition and tissue maceration, with their expression often modulated by host-derived signals [147,149].

Another pathogenicity aspect is horizontal chromosome transfer (HCT) and the role of accessory genomes where for instance, LS chromosomes harbor virulence genes and can be transferred horizontally between strains. For instance, *F. oxysporum* f. sp. *lycopersici* acquires pathogenicity via HCT of a “pathogenicity chromosome” [13,145]. Clinical *F. oxysporum* strains also carry unique LS chromosomes enriched in metal transporters, aiding niche adaptation in human hosts [145].

### 4.2. Host-Specificity and Formae Speciales in Fusarium Species

*Fusarium* species exhibit a high level of host-specificity leading to classification of strains into *formae speciales*. They can be defined as informal groups within a fungal species resulting from their ability to cause disease on specific hosts or groups of hosts. The concept of formae specialis (f. sp.) in *Fusarium* taxonomy is particularly prominent in *Fusarium oxysporum*, which comprises over 100 recognized *formae speciales*, each specialized to infect a narrow host range [150,151]. These *formae speciales* include *F. oxysporum* f. sp. *lycopersici* (tomato), f. sp. *cubense* (banana), f. sp. *vasinfectum* (cotton), and many others, each named after their primary host species or genus (Table 4). Unlike morphologically defined species, *formae speciales* are determined by host-specificity and pathogenic behavior, often without corresponding genetic divergence [152]. *Fusarium solani*, though previously categorized into multiple *formae speciales* such as f. sp. *pisi* and f. sp. *cucurbitae*, is now treated as a species complex (FSSC) due to high genetic diversity, and its *formae speciales* nomenclature is being phased out in favor of phylogenetic species designations [57,153]. In contrast, other economically important species like *F. graminearum*, *F. verticillioides*, and *F. proliferatum* exhibit broad host ranges and do not utilize *formae speciales*, as they lack the tightly host-specific infection patterns seen in *F. oxysporum* [154]. The *formae specialis* concept remains a vital, albeit imperfect, tool for understanding *Fusarium*-host interactions, particularly in pathotype delineation for resistance breeding and diagnostics.

### 4.3. Plant Immune Responses and Fusarium Counter-Defenses

The interaction between plants and *Fusarium* species is governed by a complex network of host immune responses and pathogen-derived countermeasures. Plants possess an innate immune system comprising multiple layers of defense. The first layer, pattern-triggered immunity (PTI), is initiated upon recognition of pathogen-associated molecular patterns (PAMPs) by membrane-localized pattern recognition receptors (PRRs). In the case of *Fusarium*, heat-stable, non-proteinaceous molecules derived from the fungal cell wall have been identified as PAMPs that elicit PTI in *Arabidopsis thaliana* and cotton. These molecules activate mitogen-activated protein kinase (MAPK) cascades and induce expression of defense-associated genes, thereby enhancing the plant’s basal immunity [155].

In addition to PTI, plants utilize effector-triggered immunity (ETI), a more robust and specific immune response mediated by intracellular nucleotide-binding leucine-rich repeat (NLR) proteins. ETI is activated upon recognition of pathogen effectors, leading to a localized hypersensitive response. In tomato (*Solanum lycopersicum*), resistance genes such as *I-2* and *I-3* confer immunity to *Fusarium oxysporum* f. sp. *lycopersici* through the recognition of effector proteins such as SIX3 (Avr2) and SIX1 [132]. These R genes are a product of co-evolution with the pathogen and are integral to resistance breeding programs in Solanaceous crops.

Hormonal signaling pathways involving salicylic acid (SA), jasmonic acid (JA), and ethylene (ET) further regulate plant defense responses. Each hormone plays a distinct role depending on the pathogen’s lifestyle. For instance, in *Cucumis sativus* infected with *F. oxysporum* f. sp. *cucumerinum*, transcriptomic analyses revealed significant upregulation of ET-responsive genes, highlighting the involvement of ethylene signaling in the host defense mechanism [156]. These hormonal pathways orchestrate defense responses such as reactive oxygen species (ROS) production, cell wall reinforcement, and synthesis of pathogenesis-related proteins.

Moreover, RNA silencing mechanisms contribute to plant immune regulation. Small RNAs, including microRNAs (miRNAs) and phased secondary siRNAs (phasiRNAs), modulate the expression of immune-related genes, particularly NLRs, to balance immune activation and prevent deleterious autoimmunity. This epigenetic regulation enhances the plant’s capacity to respond to rapidly evolving pathogens, including *Fusarium* species [157]. In response, *Fusarium* has evolved multiple strategies to circumvent host immunity. One major counter-defense involves the secretion of effector proteins that suppress host immune responses. For example, *F. oxysporum* f. sp. *cubense* secretes FoSSP71, a small, secreted protein that inhibits plant immunity by suppressing ROS accumulation and callose deposition, thereby promoting fungal colonization and disease development [158]. These effectors often interfere with key signaling components such as MAPK pathways, diminishing the effectiveness of both PTI and ETI [155].

Additionally, several *Fusarium* species produce an array of cell wall-degrading enzymes (CWDEs), which facilitate host tissue invasion and nutrient acquisition. These enzymes also interact with the host immune system. For instance, a pectate lyase secreted by *Fusarium sacchari* induces defense-related gene expression in *Nicotiana benthamiana*, functioning dually as both a virulence factor and a modulator of plant immunity [159]. This dual functionality underscores the complexity of host–pathogen interactions and highlights the adaptive mechanisms employed by *Fusarium*.

In conclusion, the interaction between plant immune systems and *Fusarium* species involves a dynamic and multilayered exchange of molecular signals. Understanding these mechanisms is critical for the development of resistant crop varieties and for the implementation of sustainable disease management strategies in agriculture.

## 5. Toxins Produced by Fusarium: Global Burden in Major Staple Crops

The genus *Fusarium* is currently recognized as the most prolific producer of mycotoxins within the fungal kingdom [9,160]. While several reviews have addressed *Fusarium* mycotoxins, covering their chemistry [161,162], toxicology [163,164], and the genetic regulation of their biosynthesis [165,166,167,168], less emphasis has been placed on integrating information on global and regional patterns of *Fusarium* toxin occurrence, particularly in relation to dietary exposure and regulatory oversight. This section focuses on the global burden of *Fusarium*-induced mycotoxicoses by examining their prevalence in three of the world’s most widely consumed staple crops: maize, wheat, and rice. These grains form the dietary foundation for billions of people, yet the risk of chronic exposure to *Fusarium* mycotoxins such as fumonisins, trichothecenes, and zearalenone is often underrecognized, especially in regions with weak regulatory frameworks [169]. For example, in Mexico, maize-based products like tortillas are consumed by millions of people daily, but there is limited enforcement of mycotoxin thresholds, which can lead to sustained public health risks. By evaluating regional trends in toxin occurrence alongside crop consumption patterns, this review aims to identify areas of elevated risk and underscore the need for targeted monitoring and mitigation efforts in vulnerable populations.

### 5.1. Overview of Major Fusarium-Derived Toxins in Staple Crops

Maize (also known as corn), wheat, and rice are the world’s most produced and consumed cereal crops, forming the backbone of global food security [170]. Their dominance is evident in both production volumes and harvested areas across various regions. Maize is grown globally in approximately 206 million hectares [171]. In most growing regions, maize is predominantly affected by fumonisins (especially FB_1_ and FB_2_), and by deoxynivalenol (DON) and zearalenone (ZEA), due to *Fusarium* infection in the field or during storage [160,168,172]. Wheat is the most widely grown crop worldwide and is frequently affected by trichothecenes (notably DON), nivalenol (NIV), and ZEA [173,174] (Table S1). Rice, like maize and wheat, is one of the world’s most produced and consumed cereal crops. Rice is less commonly studied than maize and wheat but is known to carry fumonisins, DON, and beauvericin in some areas [175,176].

### 5.2. Regional Analysis of Mycotoxin Burden and Exposure Risk

The occurrence and impact of *Fusarium* mycotoxins vary markedly by region due to differences in climate, crop production systems, dietary dependence on susceptible grains, and the enforcement of food safety regulations [177,178,179] (Table S2). In regions such as sub-Saharan Africa and parts of Latin America, maize is a dietary staple, yet regulatory oversight and testing infrastructure are often limited. This raises the risk of chronic exposure to fumonisins, which are frequently detected in field-infected maize [180,181]. Similarly, in parts of Asia, rice and wheat form the nutritional backbone, with reports indicating significant levels of trichothecenes like DON and T-2 toxin, particularly under poor postharvest storage conditions [182,183]. Regional disparities in exposure risk are further compounded by climatic conditions favorable to *Fusarium* proliferation such as high humidity and fluctuating temperatures which exacerbate infection in the field and toxin accumulation in storage [184]. Importantly, populations in these high-risk areas may lack awareness of mycotoxin-related health risks, and food consumed locally may bypass formal safety checks [185]. These overlapping factors create a geography of vulnerability where both mycotoxin burden and exposure risk are disproportionately high. A regionalized approach to monitoring, education, and regulation is thus essential to effectively reduce mycotoxin-related health impacts and to guide resource allocation for food safety interventions [186]. This regional analysis highlights the need for tailored strategies in monitoring, regulation, and public education to mitigate the health risks posed by *Fusarium* mycotoxins. Understanding the specific challenges and exposure risks in each region is crucial for developing effective interventions and ensuring food safety globally.

### 5.3. Mycotoxin Detection

Recent reviews have comprehensively summarized the state of mycotoxin detection, highlighting both established and emerging techniques. Chromatographic methods such as HPLC and GC-MS remain the gold standard due to their high sensitivity and specificity, particularly for regulated mycotoxins like deoxynivalenol (DON), zearalenone (ZEN), and fumonisins [187]. Immunoassays, especially ELISA, continue to be widely used for rapid screening, offering cost-effective and high-throughput options despite occasional cross-reactivity and matrix interference [188]. Meanwhile, molecular diagnostics like qPCR and LAMP are gaining traction for early detection of *Fusarium* DNA and mycotoxin biosynthesis genes, enabling preemptive management before toxin accumulation [189].

Recent advances also include biosensor technologies, aptamer-based assays, and machine learning-assisted predictive models that aim to improve detection speed and accuracy [188]. These innovations are particularly promising for multiplex detection and on-site diagnostics. However, despite these developments, a critical gap remains in the detection of masked and emerging mycotoxins, which are often overlooked by conventional assays. These compounds may escape detection due to their altered chemical structures or low concentrations, yet they can still pose significant health risks once metabolized in humans or animals [187,189].

Moreover, the lack of standardized protocols for sample preparation, especially in heterogeneous matrices like silage or processed feeds, continues to hinder reproducibility and comparability across laboratories [188]. Addressing this gap will require harmonized validation frameworks and broader adoption of integrative platforms that combine molecular, immunological, and chemical detection methods. As the field evolves, the integration of high-resolution mass spectrometry with real-time biosensing and AI-driven analytics holds promise for more comprehensive and proactive mycotoxin surveillance.

## 6. Management and Diagnostic Strategies of Fusarium-Induced Plant Diseases

Managing *Fusarium*-induced plant diseases requires a multifaceted approach that integrates traditional, biological, molecular, and advanced methods. Conventional methods such as crop rotation and chemical fungicide applications remain essential in limiting disease spread, although concerns over sustainability and resistance drive the search for alternative solutions. Biocontrol methods, including microbial antagonists and endophytic fungi, show promise in suppressing *Fusarium* pathogens through natural competition and protective mechanisms. Molecular diagnostics, such as qPCR, LAMP, and nanopore sequencing, enable rapid and accurate detection of *Fusarium* species, improving disease surveillance and early intervention. Breeding for resistance has seen notable successes but continues to face challenges due to genetic variability among *Fusarium* strains; novel tools like CRISPR offer potential breakthroughs for precise resistance gene editing. Looking ahead, predictive modeling enhances disease forecasting, while RNAi-based strategies and integrated pest management (IPM) pave the way for innovative, sustainable disease control solutions. A combination of these approaches offers the best path toward effective management and long-term protection of susceptible crops.

Several practices including cultural, such as crop rotation, along with modern approaches, such as chemical interventions, have long been the mainstays of management strategies for various *Fusarium*-induced diseases [190,191,192]. In the next couple paragraphs, we discuss some of the successful management approaches for *Fusarium*-induced diseases.

(a) Crop Rotation: Prior to the widespread use of synthetic fertilizers and pesticides in the 1950s, crop rotation played a particularly important role in the management of diseases and pests [193]. Because *Fusarium* inoculum is widely distributed in soil, on plant parts, and on debris, crop rotation has been the foundational basis for limiting its accumulation [194,195]. Crop rotation lowers the risk of disease development and pressure by using a nonhost crop to disrupt a *Fusarium species’* life cycle [196,197,198].

However, crop rotation might not always be a successful disease control strategy because many *Fusarium* species have wide geographical and host ranges. Several significant species of *Fusarium*, including *F. graminearum* and *F. oxysporum*, have been isolated from corn, soybean, pea, chickpea, lentil, wheat, sorghum, and canola [7,194,199,200,201,202,203,204,205]. Furthermore, the ability of *Fusarium* pathogens to survive for long periods, sometimes up to 10 years, as chlamydospores in the soil, complicates the long-term efficacy of crop rotation [206,207,208].

Studies have demonstrated that the impact of crop rotation on *Fusarium* populations is species-specific. For instance, Marburger et al. [191] reported that crop rotation had little to no effect on the soil populations of *F. oxysporum* and *F. virguliforme* populations. In contrast, *F. graminearum* was detected more frequently in continuous wheat plots (44%) compared to those under corn—soybean—wheat rotation (13%). These findings suggest that while crop rotation may impact certain *Fusarium* species, its effectiveness varies depending on the biology of the pathogen and environmental factors rather than offering uniform control across the genus [209].

(b) Chemical Control: The use of fungicides remains a common component of integrated management strategies aimed at mitigating diseases caused by *Fusarium* species. In large-scale cropping systems, this approach is often favored for its efficiency, ease of application, and relatively rapid suppression of disease symptoms [210]. The choice of fungicide depends on the species and the disease location—aboveground (foliar fungicide application) or soil/stubble borne (seed treatment) [145,191,211,212,213].

Several studies have evaluated the efficacy of foliar fungicides for controlling aboveground diseases such as Fusarium Head Blight (FHB) caused by *Fusarium graminearum* in cereals and reducing associated mycotoxin accumulation (e.g., Deoxynivalenol—DON). However, the observed efficacy has varied from substantial suppression to insignificant impact [214,215,216,217,218,219,220].

Fungicide seed treatments are commonly used to manage root-infecting pathogens such as those causing sudden death syndrome (SDS). This approach is often favored for the ineffectiveness of foliar fungicides [191,221]. The effectiveness of seed treatments is influenced by the *Fusarium* species and the fungicide’s mode of action [213,222,223].

Several potentially effective fungicides against *Fusarium* species are applied as either seed treatments or foliar sprays include azoxystrobin, carbendazim, cyclobutrifluram, difenoconazole, fludioxonil, fluopyram, phenamacril, prothioconazole, pyraclostrobin, tebuconazole, and trifloxystrobin [210,224,225,226] (Table 5).

While these chemical fungicides are considered effective to some degree, their overall field performance is influenced by variable weather conditions and the level of pathogen resistance. The emergence of fungicide-resistant *Fusarium* strains due to frequent and indiscriminate use poses a challenge to their efficacy [210,213,227,228,229] and raises environmental and human health concerns [230,231,232,233]. Therefore, there is a need to integrate fungicide applications with other sustainable *Fusarium* disease management strategies such as biological control to enhance their efficacy and minimize the risk of resistance development.

**Table 5:** Some commonly used fungicides against diseases caused by *Fusarium species* and their mode of application.

Fungicide (Common Name)	Commercial Name (If Specificied)	FRAC Category/Chemical Group	Mode of Application	Target *Fusarium* Species/Disease	References
Azoxystrobin	Azimut, Amistar, Dynasty, Ortiva	Strobilurin (QoI, FRAC group 11)	Seed coating, Culture plates, Seed treatment, Foliar spray, in vitro	*F. acutatum*, *F. oxysporum* f. sp. cepae, *Fusarium* spp., *F. virguliforme*, *F. subglutinans*, *F. temperatum*, *F. graminearum*, *F. oxysporum*, *F. pseudograminearum*	[200,234,235,236]
Carbendazim	Antracol	Benzimidazole (Systemic, Broad-spectrum)	Seed treatment, Soil mixture, Culture media	*Fusarium* spp., *F. oxysporum* f. sp. *vasinfectum*, *F. oxysporum* f. sp. lentis, *F. oxysporum* (maize), *F. equiseti*, *F. chlamydosporum*, *F. pseudograminearum*	[227,228,229,230,231,232,233,234,235,236,237,238,239]
Cyclobutrifluram	-	SDHI (FRAC group 7)	In vitro	*F. pseudograminearum*	[240]
Difenoconazole	Dividend XL RTA	Azole (DMI)	Culture plates, Seed treatment, in vitro	*F. solani*, *F. proliferatum*, *F. oxysporum*, *F. circinatum*, *F. avenaceum*, *F. culmorum*, *F. poae*, *F. sporotrichioides*, *F. subglutinans*, *F. temperatum*, *F. pseudograminearum*	[241,242,243,244]
Fludioxonil	Vibrance, Maxim 4 FS, MaximQuattro, Celest XL, Celest Quattro	Phenylpyrrole	Seed coating, Culture plates, Seed treatment	*F. acutatum*, *F. oxysporum* f. sp. *cepae*, *Rhizoctonia*, *Fusarium* spp., *F. graminearum*, *F. virguliforme*, *F. solani*, *F. oxysporum* (dry rot), *Sclerotinia sclerotiorum*, *F. verticillioides*, *F. pseudograminearum*	[200,213,223]
Fluopyram	ILeVO	SDHI (FRAC group 7)	Seed treatment	*F. virguliforme*	[221]
Phenamacril	-	Myosin inhibitor	In vitro	*F. pseudograminearum*	[226,240]
Prochloraz	Sportak	Azole (DMI)	Seed coating, Potted sprout irrigation, Culture plates	*F. oxysporum* f. sp. *cepae*, *F. acutatum*, *F. subglutinans*, *F. temperatum*, *F. oxysporum* (banana wilt), *F. graminearum* (FHB), *F. culmorum* (FHB), *F. oxysporum* f. sp. *lycopersici*, *F. pseudograminearum*	[235,244]
Prothioconazole	Redigo, Proline, Prosaro	Azole (DMI, FRAC group 3)	Seed treatment, Foliar fungicide, in vitro	*Fusarium* spp., *F. virguliforme*, *F. graminearum* (FHB), *F. poae*, *F. pseudograminearum*	[222,245,246]
Pyraclostrobin	Stamina, BAS 580	Strobilurin (QoI, FRAC group 11)	Seed treatment, Foliar fungicide, in vitro	*Fusarium* spp., *F. virguliforme*, *F. graminearum*, *F. pseudograminearum*	[223,240,245]
Tebuconazole	Orius 25, Azimut, Raxil 250 FL, Raxil MD, Raxil T, Folicur, Nativo SC300, Twinstar 75 WG, Prosaro	Azole (DMI, FRAC group 3)	Culture plates, Seed coating, In vitro, Foliar spray	*F. acutatum*, *F. oxysporum* f. sp. *cepae*, *F. subglutinans*, *F. temperatum*, *F. graminearum* (FHB), *F*. *culmorum* (FHB), *F. poae*, *Fusarium equiseti*, *F. chlamydosporum*, *Fusarium* spp., *F. pseudograminearum*	[243,244,247]
Thiabendazole	Mertect 340F, Rival, Tecto, MaximQuattro, Trilex AL (part of)	Benzimidazole (FRAC group 1)	Culture plates, Seed treatment, In vitro, In vivo, In situ	*F. solani*, *Fusarium* spp., *F. oxysporum*, *F. graminearum (part of combination)*, *F. verticillioides* (part of combination)	[241,248]
Trifloxystrobin	Trilex, Fortix	Strobilurin (QoI, FRAC group 11)	Seed treatment, Foliar fungicide, in vitro	*Fusarium* spp., *F. graminearum*, *F. virguliforme*, *F. chlamydosporum*, *F. asiaticum*	[243,247,249]

The effectiveness of fungicides varies significantly depending on the specific *Fusarium* species, isolate, environmental conditions, and the mode of action. Resistance can emerge, particularly with single-site fungicides such as those in the Qol and DMI groups [210,250]. Disclaimer: The fungicide active ingredients listed in Table 5 are included based on published studies evaluating their efficacy against *Fusarium* spp., either alone or in combination with other products. Efficacy may vary depending on crop, pathogen species, environmental conditions, and formulation. Some compounds (e.g., Azoxystrobin and Metalaxyl) have shown limited or inconsistent activity against *Fusarium* and are typically included in seed treatment packages for their broad-spectrum or oomycete-targeted action rather than direct control of *Fusarium*. Their inclusion here reflects their presence in commercially available mixtures and literature reports, not a recommendation of high standalone efficacy.

(c) Biocontrol: This offers a sustainable alternative to chemical fungicides for the management of plant diseases caused by *Fusarium species*, reducing the impact of associated environmental and human health risks [251,252]. Several biological control agents (BCAs), including strains of *Trichoderma* species and *Bacillus velezensis* have demonstrated efficacy in suppressing *Fusarium*-induced plant diseases across both controlled and field environments [251,253,254,255].

The BCAs employ different mechanisms to inhibit *Fusarium species*, such as: (1) competition for nutrients and or space. For instance, strains of *Bacillus* and *Pseudomonas* sequester iron through siderophores, thereby limiting the growth of fungal pathogens [256]. (2) Production of antifungal metabolites by genera like *Trichoderma* and *Bacillus* with potent antifungal activity [257,258]. (3) Induction of systemic resistance of host plant defenses, enhancing defense against Fusarium infections [256,257,259]. (4) Disruption of pathogen life cycles. BCAs, like *Clonostachys rosea*, are well-known antagonists that can colonize crop residues, inhibiting the reproductive structures like the perithecia [260,261,262].

Non-pathogenic *Fusarium* strains, like *F. oxysporum* Fo47 and CS-20, and fungal/bacterial endophytes are also known to function as effective BCAs [263,264,265,266]. For example, in melon and tomato, they compete for space and nutrients and induce host resistance to significantly reduce the disease incidence [267]. Some endophytes show specific adaptability due to their shared niche with internal pathogens and close host relationships, thereby enhancing their persistence under field conditions [268,269,270,271].

For reliable field efficacy, the selection of BCAs must be prioritized on strains with environmental resilience, compatibility with formulation processes, and integrated into the broader disease management strategies [272,273,274].

(d) Use of Resistant Cultivars: Host resistance remains one of the most effective and sustainable *Fusarium* management strategies. Conventional breeding has identified several quantitative trait loci (QTLs) conferring resistance to *Fusarium* diseases, like FHB in wheat and barley, and Fusarium wilt in grain legumes [275,276,277,278]. However, conventional breeding methods are labor-intensive, can take a long time, and require enough genetic variation in the breeding material [279,280].

Recent advancements in molecular biology and genomic technologies, such as marker-assisted selection (MAS) and genomic selection (GS), have accelerated the introgression of resistance genes into elite lines and the development of new cultivars [279,280,281,282]. MAS has been successfully applied to select for large-effect QTLs and choose the prominent resistance QTL *Fhb1* in bread wheat and durum wheat [283,284]. Furthermore, in crops like rice, wheat and maize, successful application of MAS has improved resistance against diseases such as bacterial blight, blast, rusts, and northern corn leaf blight [285].

Genomic selection, a breeding method which employs genome-wide prediction models, has been used to predict with precision mycotoxin accumulation, thus improving the accuracy of resistant cultivar selection and potentially reducing the breeding cycles [286]. Genomic selection has shown promise for breeding programs aimed at FHB resistance [287].

Despite the breeding progress, the success of marker-assisted breeding depends on several variables such as marker quality, the genetic basis of traits, linkage-related genes, and their effects [288]. In addition, polygenic resistance and genotype-environment interactions pose major challenges, while the cost for genome-wide marker coverage, high-throughput, and cost-efficient phenotyping technologies remains high. To this end, genome editing (GE) and clustered regularly interspaced short palindromic repeats (CRISPR)/CRISPR-associated endonuclease 9 (Cas9) hold promise for revolutionizing plant breeding for disease resistance [282,289].

Genome editing methods like CRISPR/Cas9, transcription activator-like effector nucleases (TALENs), and zinc-finger nucleases (ZFNs) are a transformative approach to develop resistant varieties with high precision and affordability [290,291,292]. By directly editing trait-responsive genes in elite breeding lines or commercial cultivars, linkage drag, and time-consuming, labor-intensive backcrossing can be avoided [293]. CRISPR/Cas has been successfully used to improve resistance against various bacterial and fungal diseases [291,294,295,296,297,298]. In their study, Brauer et al. [299] showed that CRISPR-mediated editing of wheat susceptibility genes significantly reduced the severity of the disease, suggesting that targeted genome editing of susceptible genes can be an effective strategy for enhancing wheat disease resistance.

Although genome editing is a powerful and promising tool for developing disease-resistant crops, its full potential can only be realized when combined with conventional breeding approaches [300]. This would ensure that the crop varieties produced are both genetically diverse and well-adapted to real-time growing conditions [282,301].

(e) Predictive modeling, RNAi, and integrated disease management approaches:

The next frontier in the strategic management and control of *Fusarium*-induced diseases involves combining advanced technologies with ecological insights. This requires implementation of future-forward approaches that leverage predictive tools, innovative biotechnologies like RNA interference (RNAi), and comprehensive integrated pest management (IPM) principles.

Predictive Modeling: *Fusarium* species cause major crop diseases such as blight, root and stem rots, and wilts across various climatic zones [301,302]. Understanding how common species of *Fusarium*, like *F. graminearum* and *F. oxysporum*, are likely to respond to changing climatic conditions is essential for timely interventions to minimize crop losses [302,303].

Predictive tools such as species distribution models (SDMs) help give early warnings on fungal growth and spread under different climatic conditions [302]. Models like generalized linear models (GLM), maximum entropy (MaxEnt), and generalized boosting models (GBM), together with climate variables like temperature and rainfall, can be used to predict the habitats and spread of fungal pathogens [301,302].

Ensemble predictions, a technique combining outputs from multiple SDMs, help minimize uncertainty and generate more reliable projections on how specific *Fusarium* species will evolve under different climate change scenarios [302,304]. This provides an opportunity for proactive strategic management methods.

Therefore, predictive modeling based on weather, crop phenology, and pathogen biology enables early warnings and helps reduce yield losses [305]. These models have already predicted higher risks of diseases linked to *Fusarium* in Asia, Europe, the Americas, and Australia [302]. De Wolf et al. [306] developed risk models that guide fungicide application in wheat for FHB caused by *F. graminearum*. Thus, integrating such models with real-time data can optimize intervention timing.

RNA interference (RNAi): These approaches are a natural process that controls gene expression via small RNA (sRNA) molecules, resulting in sequence-specific gene silencing [303,304,307,308,309]. They have emerged as promising, environmentally safe management strategies against insect pests and fungal pathogens, including *Fusarium* species [303]. In RNAi approaches, genes necessary for growth, development, or pathogen virulence can be specifically silenced [303,307]. There are two key RNAi-based strategies for crop protection. These include Host-Induced Gene Silencing (HIGS) and Spray-Induced Gene Silencing (SIGS) [307,308].

In HIGS, plants are genetically modified to generate double-stranded RNAs (dsRNAs) that target important fungal genes [308,310]). During infection, these dsRNAs are converted into small interfering RNAs (siRNAs), which enter the invading *Fusarium* cells and silence genes essential for virulence and fungal growth [307,308,309,310,311]. HIGS has been used to target key genes such as the CYP51 gene family in *F. graminearum*, which are targets of azole fungicides, enabling synergistic or alternative control measures [310].

In contrast, SIGS represents a non-transgenic alternative that involves spraying plants with dsRNA or siRNA molecules aimed at silencing specific pathogen genes [307,308,311,312,313].

The pathogen may directly absorb these external RNA molecules from the plant surface, or the plant may absorb and transfer them to the pathogen. By focusing on genes involved in fungal development and toxin production, studies have shown SIGS to reduce disease in crops such as tomato and barley [308,312,314]. However, RNA stability in environmental settings remains a challenge, and current studies are looking into the use of stabilizers and nanoparticles to increase RNA uptake and longevity [159,307,315]. A SIGS-based dsRNA product’s commercial registration against the Colorado potato beetle demonstrates the technology’s usefulness and potential for more extensive uses in fungal disease control, such as those caused by *Fusarium* species [311].

While RNAi approaches offer advantages over traditional chemical treatments due to their sequence specificity, challenges remain. These include the variability in RNAi effectiveness among *Fusarium* species and the potential for resistance development over time [307,316]. To address these challenges, it is important to include resistance management strategies like using multiple targets or combining RNAi with other complementary control methods [307,316].

Integrated Pest Management (IPM): IPM involves a combination of complementary management strategies to reduce disease pressure and enhance long-term agricultural sustainability. The effective management of diseases caused by *Fusarium* species should involve an integrated approach, combining agronomic practices, use of resistant cultivars, chemical control, and biocontrol (as previously discussed). This is important due to the complex population biology and genetic diversity of *Fusarium* species [88].

Integrating predictive modeling, RNAi technologies, host resistance, cultural practices, chemical use, and biological controls will form a powerful, multifaceted approach to Fusarium management and control [197,305,317].

Predictive models guide the timing of interventions such as application of fungicides, BCAs, or potentially SIGS treatments [305,317]. RNAi, on the other hand, with its species-specific targeting, offers an exact tool that can be integrated into existing spray programs (SIGS) or genetic resistance strategies (HIGS) [307,310,317]. For efficient and sustainable fungal pathogen management, the development of RNAi-based biopesticides and their integration into IPM programs are essential [317]. Developing climate-smart pathogen control solutions and enhancing global food security require an integrated approach that considers climate change projections and make use of both cutting-edge biotechnologies and well-established practices [197,302,305,307]. Furthermore, incorporating advanced technologies like sensors, drones, and AI-based analytics may improve precision in managing *Fusarium* diseases.

Key Components of Integrated Disease Management (IDM): Effective management of *Fusarium*-induced plant diseases requires a multifaceted, integrated approach that combines cultural, biological, chemical, and molecular strategies. Host resistance remains a cornerstone of IDM, offering long-term and environmentally sustainable control when resistant cultivars are available. Crop rotation with non-host-species reduces soil inoculum levels, although its effectiveness may be limited by the wide host range and persistence of *Fusarium* in soil. Biological control, using antagonistic microbes such as *Trichoderma* spp. and beneficial endophytes, can suppress *Fusarium* through mechanisms like competition, antibiosis, and induced systemic resistance. The judicious application of fungicides, including triazoles and strobilurins, contributes to disease suppression but must be managed carefully to prevent resistance development. Molecular diagnostics, such as qPCR and LAMP assays, enable early and accurate detection of *Fusarium* species, allowing timely interventions. Integrating these strategies within a crop- and region-specific framework enhances their collective efficacy and contributes to sustainable disease management while minimizing environmental and economic risks.

### Molecular Diagnostics

Rapid and correct identification of pathogens is a prerequisite for any successful disease management strategies. Traditional pathogen diagnostic methods, such as culturing and morphological observation, are often slow and unreliable, particularly for non-culturable pathogens. Traditional identification of *Fusarium*, which comprises many species that have seen numerous revisions since their inception, has been historically accomplished by using several manuals detailing essential synoptic keys [195,318,319,320]. The recent development of FusaHelp, an open-access, web-assisted resource platform, has significantly streamlined conventional *Fusarium* identification [16].

While traditional methods remain in use, molecular techniques, including PCR of specific marker genes like TEF, rpb2, DNA hybridization, and sequencing, have significantly improved the speed and cost-effectiveness of diagnosis for a huge number of *Fusarium* species of agronomic concern [321,322] and of human disease relevance [33]. The most innovative achievements in the field of identification of *Fusarium* speciesinvolve the development of techniques even more rapid, precise, high throughput, economic and possibly portable.

Among the most notable innovations is Digital PCR (dPCR), which enables highly sensitive and absolute quantification of DNA by partitioning the reaction mix into thousands of individual compartments [323]. dPCR has been effectively applied to quantify trichothecene-producing *Fusarium* species [324,325] and *F. oxysporum* f. sp. *cubense* Tropical Race 4 (Tr4) [326,327]. Compared to qPCR, dPCR offers greater precision at low target concentrations, is less affected by inhibitors, and does not require reference standards. Another important innovation is the availability of new technology that can amplify DNA at constant and low temperatures thus eliminating the requirement for thermal cyclers. Loop-mediated isothermal amplification (LAMP) has become popular for pathogen detection and disease diagnosis. It requires four to six primers to synthesize DNA amplicons of various sizes [328,329].

Among others, positive applications of LAMP have been achieved for the rapid detection of *F. oxysporum* f. sp. *ciceris* [330], and of *F. fujikuroi* on rice seeds [331]. Another isothermal nucleic acid amplification technique is the Recombinase Polymerase Amplification (RPA), in which 30 to 35 bp-long primers form a complex with a recombinase enzyme, which binds to homologous DNA regions resulting in the formation of D-loop structures. It requires only one pair of oligonucleotide primers, and RPA amplicons using an oligonucleotide probe can be visualized in real time using lateral flow dipsticks (LFDs) [332] also halving the reaction time as compared to LAMP. It has been utilized for the detection of *F. asiaticum* [54], *F. oxysporum* [333], and *F. graminearum* [334].

Technological progress in high-throughput sequencing (HTS), with new sequencers from companies like Illumina, PacBio, and Oxford Nanopore and the development of data analysis pipelines, has revolutionized diagnostics by enabling rapid and cost-effective sequencing of numerous microorganisms, which aids in monitoring diseases through environmental DNA (eDNA) analysis and decreases dependence on taxonomic expertise. These techniques have been utilized for the diagnosis of *Fusarium* species in complex environments such as soil [335] or of complex etiology diseases like Fusarium Head blight [336,337,338].

Fungi produce hundreds of volatile organic compounds (VOCs) as a byproduct of their metabolism [339]. These compounds are responsible for different smells that could be utilized for the early detection of fungi in plants or in transformed commodities [340]. The study of VOCs (*volatome* or *volatilome*) is an emerging field intersecting with medicine, agriculture, and ecology [341]. Analytical methods for studying VOCs include PTR-ToF-MS (Proton-Transfer-Reaction Time-of-Flight Mass Spectrometry), SPME-GC/MS (Solid-Phase Microextraction Gas Chromatography/Mass Spectrometry), and electronic nose. They are fast and sensitive for detecting trace and low-molecular-weight VOCs, require minimal samples, no solvents, and no pre-treatment, thus preventing sample alteration. Several examples of practical applications of VOCs produced by *Fusarium* species are available, particularly for the rapid detection of species involved in FHB and relative mycotoxins [342,343,344] or other species, i.e., rice [345], onions [346], maize [347], and garlic [348].

These techniques will be relevant for the development of portable diagnostic technologies, including handheld biosensors and smartphone platforms, supporting advanced diagnostic assays in field settings, facilitating precision agriculture and evidence-based decision-making while integrating with Internet of Things (IoT) and cloud systems.

## 7. Conclusions and Future Perspectives

The battle against *Fusarium* is far from over. As we continue to unlock its genomic complexity and refine its taxonomy, the path forward must integrate molecular insights with field-tested management strategies. The goal is not eradication, which may be unrealistic, but rather a sustainable equilibrium that balances scientific innovation with ecological stewardship and practical disease control.

This review highlights the remarkable adaptability and diversity of the *Fusarium* genus, which continues to challenge traditional paradigms in plant pathology, taxonomy, and disease management. The emergence and redefinition of species complexes such as FOSC, FSSC, FGSC, FFSC, FTSC, and FIESC underscore the need for integrative taxonomic frameworks that accommodate cryptic diversity, ecological plasticity, and host specificity [17,32]. The exclusion of other important complexes such as *Fusarium sambucinum* species complex (FSAMSC), *Fusarium chlamydosporum* species complex (FCSC), and *Fusarium dimerum* species complex FDSC from many diagnostic and surveillance systems represents a critical gap that must be addressed to ensure comprehensive pathogen monitoring and risk assessment.

Advances in multi-omics particularly genomics, transcriptomics, and proteomics have revolutionized our understanding of *Fusarium* pathogenicity, host adaptation, and mycotoxin biosynthesis [13,44]. However, the translation of these insights into practical tools for disease forecasting, resistance breeding, and biocontrol remains uneven. The development of portable diagnostics, real-time surveillance systems, and predictive models tailored to regional agroecosystems will be essential in bridging this gap [16].

The emergence of *Fusarium* pathogens in niche crops and underexplored geographies, driven by climate change, global trade, and agricultural intensification, signals a broader ecological shift [71,76]. These trends demand a proactive, globally coordinated response that integrates pathogen genomics, crop-specific management strategies, and international phytosanitary policies.

Looking ahead, the convergence of CRISPR-based genome editing, RNA interference (RNAi), and AI-driven analytics offers unprecedented opportunities to engineer durable resistance, optimize disease management, and anticipate pathogen evolution [299,308]. Yet, these innovations must be grounded in equitable access, regulatory clarity, and farmer-centered implementation to ensure their global impact.

In conclusion, the future of *Fusarium* research lies in embracing complexity biological, ecological, technological, and fostering interdisciplinary collaboration. By doing so, we can transform our understanding of this challenging genus into actionable strategies that safeguard global food security, environmental health, and agricultural resilience.

## Figures and Tables

**Table 2:** Key transcription factors regulating host-specific pathogenicity and secondary metabolism in *Fusarium* species.

Transcription Factor	*Fusarium* Species	Function	Regulates	References
Tri6	*F. graminearum*	Zn(II)2Cys6 TF; pathway-specific regulator	Trichothecene biosynthesis gene cluster (TRI genes); also acts as global regulator	[122,123]
Tri10	*F. graminearum*	Transcriptional activator	Activates TRI gene expression in coordination with Tri6	[122]
PacC	*F. oxysporum*	pH-responsive TF	Virulence gene expression under neutral-alkaline pH; adaptation to host environment	[124]
Ftf1	*F. oxysporum*	Zn(II)2Cys6 TF encoded on accessory chromosome	Effector gene expression and host-specific pathogenicity	[125]
Sge1	*F. oxysporum*	Global TF involved in effector regulation	SIX gene expression (secreted in xylem proteins)	[126]
FgLaeA (part of VeA complex)	*F. graminearum*	Global regulator of secondary metabolism, sexual development, and virulence	Regulates the expression of trichothecene biosynthetic genes (TRI6, ZEB2), sexual development genes, and virulence factors via interaction with FgVeA	[127]
Kmt6 (H3K27 methyltransferase)	*F. fujikuroi*	Epigenetic regulator of secondary metabolism via histone modification	Controls the activation of silent secondary metabolite gene clusters by modifying chromatin accessibility through H3K27me3 marks	[128]
AreA	*F. fujikuroi*	Nitrogen metabolism TF	Gibberellin biosynthesis genes (in response to nitrogen availability)	[129]

**Table 3:** Economically important *Fusarium* species, major host crops, and associated resistance genes.

*Fusarium* Species	Host Crop	Disease	Resistance Gene/Locus	References
*F. graminearum*	Wheat	Fusarium head blight	Fhb1	[133]
*F. oxysporum* f. sp. *lycopersici*	Tomato	Fusarium wilt	I I-2 I-3	[134]
*F. oxysporum* f. sp. *lentis*	Lentil	Fusarium wilt/root rot	Fw Gene	[135,136]
*F. virguliforme (syn. F. solani* f. sp. *Glycines)*	Soybean	Sudden death syndrome	Rhg1 Rhg4	[137]
*F. verticillioides*	Maize	Ear rot, seedling blight	ZmWAX2 ZmXYXT2	[138,139]
*F. oxysporum* f. sp. *cubense* tropical race 4 (Tr4)	Banana	Fusarium wilt (Panama disease)	QTLs (Ma848 and Ma851)	[140]
*F. oxysporum* f. sp. *vasinfectum*	Cotton	Fusarium wilt	GhWAK7A	[141]
*F. oxysporum* f. sp. *cucumerinum*	Cucumber	Fusarium wilt	QTLs CsChi23	[142,143]

Note: Resistance genes listed here are representative, not exhaustive. f. sp. = *forma specialis*.

**Table 4:** *Formae speciales* status of some of the common *Fusarium* species.

*Fusarium* Species	Known for *Formae speciales*?	Notable *Formae speciales* (f. sp.)
*F. oxysporum*	Yes—over 100	f. sp. *lycopersici* (tomato), *cubense* (banana), *vasinfectum* (cotton), *cepae* (onion), *melonis* (melon), *pisi* (pea)
*F. solani* (FSSC)	Yes, but being revised	f. sp. *pisi* (pea), *phaseoli* (bean), *cucurbitae* (cucurbits), *mori* (mulberry)
*F. graminearum*	No	Not applicable—broad host range
*F. verticillioides*	No	Not applicable—infects maize, sorghum, and others broadly
*F. proliferatum*	No	Not applicable—opportunistic across many hosts
*F. avenaceum*	No	Not applicable—generalist necrotrophy

## Supplementary Materials

The following supporting information can be downloaded at https://www.mdpi.com/article/10.3390/pathogens14080762/s1, Table S1: Major *Fusarium* mycotoxins in staple crops and associated health effects.; Table S2: Regional overview of Fusarium-derived toxins in staple crops: Regulatory and public health concerns. The references cited in the supplementary material file can be found at [349,350,351,352,353,354,355,356,357,358,359,360,361].
