# Supplementary material for: From Morphology to Multi-Omics: A New Age of Fusarium Research
Source: Pathogens. 2025 Aug 1;14(8):762. doi: 10.3390/pathogens14080762 (PMC12389138; doi:10.3390/pathogens14080762)
Supplement: Supplementary file 1 [file pathogens-14-00762-s001.zip › pathogens-3758163-supplementary.pdf]

Table S1: Major Fusarium mycotoxins in staple crops and associated health effects.

**Mycotoxin**	**Associated Crop(s)**	**Major Fusarium Producers**	**Primary Health Effects**	**IARC* Classification**	**References**
Fumonisins (esp. FB1)	Maize	*F. verticillioides, F. proliferatum*	Neural tube defects, esophageal cancer, liver & kidney toxicity	Group 2B (possible carcinogen)	[349-351]
Deoxynivalenol (DON / vomitoxin)	Wheat, Maize	*F. graminearum, F. culmorum*	Nausea, vomiting, immune suppression, impaired growth	Not classified	[352-354]
Zearalenone (ZEA)	Maize, Wheat	*F. graminearum, F. culmorum*	Estrogenic effects: reproductive disorders, infertility, precocious puberty	Group 3 (not classifiable)	[349,355]
T-2 and HT-2 toxins	Wheat, Barley (less common in maize)	*F. sporotrichioides, F. poae*	Immunosuppression, gastrointestinal lesions, skin necrosis	Group 3	[356,357]
Nivalenol (NIV)	Wheat, Maize	*F. graminearum, F. culmorum*	Hematotoxicity, immunotoxicity	Not classified	[353,358]
Beauvericin	Maize, Rice (in some regions)	*F. proliferatum, F. subglutinans*	Cytotoxicity, ionophoric activity (mitochondrial effects)	Not classified	[359,360]
Moniliformin	Maize	*F. proliferatum, F. subglutinans*	Cardiotoxicity, skeletal defects (in animals)	Not classified	[360,361]

Notes: *IARC = International Agency for Research on Cancer. The IARC classifications indicate the strength of evidence for carcinogenicity in humans: Group 1: Carcinogenic; Group 2A: Probably carcinogenic; Group 2B: Possibly carcinogenic; Group 3: Not classifiable. Health effects listed are based on chronic dietary exposure and do not necessarily reflect acute toxicity.

Table S2. Regional overview of Fusarium-derived toxins in staple crops: Regulatory and public health concerns

**Region**	**Crops**	**Key Fusarium-derived Toxins**	**Regulatory Notes and/or Public Health Concern**
Latin America	Maize, with significant wheat and increasing rice consumption	Fumonisins in maize; occasional ZEA and DON in wheat	Varying levels of regulation; CODEX standard exist but are not uniformly adopted
Sub-Saharan Africa	Maize is dominant, with wheat and rice also present	High levels of fumonisins and DON reported; exposure risk is compounded by poor-harvest practices	High reliance on maize with limited food safety monitoring leads to elevated risk of chronic exposure and potential esophageal cancer linkage.
South and Southeast Asia	Rice is the staple crop, followed by increasing maize and wheat consumption	Occasional detection of fumonisins and DON in rice; wheat affected by trichothecenes	The regulatory environment: Some countries (e.g., India) have national standards, but enforcement varies.
Europe	Wheat is the primary staple; maize is significant in animal feed	Frequent detection of DON and ZEA in wheat; strong monitoring system exists.	Exposure Risk: Lower compared to other regions due to strict EU regulations and surveillance.

North America	Both maize and wheat are widely produced and consumed	Regular monitoring reveals fumonisins in maize, DON and ZEA in wheat	Mitigation: effective surveillance and strict FDA limits reduce exposure risk.
Middle East and North Africa	Wheat dominates, with increasing maize and rice imports	Limited data: but DON and ZEA are of concern in wheat imports.	Challenges: food insecurity and import reliance complicate mycotoxins management.
